# Trends in Mortality From Co-Occurring Diabetes Mellitus and Pneumonia in the United States (1999-2022): Retrospective Analysis of the Centers for Disease Control and Prevention Wide-ranging Online Data for Epidemiologic Research (CDC WONDER) Database

**DOI:** 10.2196/78001

**Published:** 2025-08-07

**Authors:** Asad Zaman, Ali Shan Hafeez, Abdul Rafae Faisal, Muhammad Faizan, Mohammad Abdullah Humayun, Abdullah, Mavra Shahid, Pramod Singh, Rick Maity, Arkadeep Dhali

**Affiliations:** 1 Combined Military Hospital Multan Institute of Medical Sciences, Multan, Pakistan; 2 Hamad Medical Corporation, Doha, Qatar; 3 Shalamar Institute of Health Sciences, Lahore, Pakistan; 4 Rawalpindi Medical University, Rawalpindi, Pakistan; 5 Akhtar Saeed Medical and Dental College, Lahore, Pakistan; 6 Barhabise Primary Health Care Centre, Barhabise, Sindhupalchowk, Nepal; 7 School of Medical Science and Technology, Indian Institute of Technology Kharagpur, Kharagpur, India; 8 Sheffield Teaching Hospitals NHS Foundation Trust, Sheffield, United Kingdom; 9 School of Medicine and Population Health, University of Sheffield, Sheffield, United Kingdom; 10 Deanery of Clinical Sciences, University of Edinburgh, 49 Little France Crescent, Edinburgh, EH16 4SB, United Kingdom, 44 7471323814; 11 School of Medicine, University of Leeds, Leeds, United Kingdom

**Keywords:** Centers for Disease Control and Prevention Wide-ranging Online Data for Epidemiologic Research, CDC WONDER, diabetes mellitus, pneumonia, mortality trends, United States

## Abstract

**Background:**

Pneumonia is the most common respiratory tract infection among patients with diabetes, affecting individuals across all age groups and sexes.

**Objective:**

This study aims to examine demographic trends in mortality among patients diagnosed with both diabetes mellitus (DM) and pneumonia.

**Methods:**

Deidentified death certificate data for DM- and pneumonia-related deaths in adults aged 25 years and older from 1999 to 2022 were obtained from the Centers for Disease Control and Prevention Wide-ranging Online Data for Epidemiologic Research (CDC WONDER) database. Age-adjusted mortality rates (AAMRs) per 1,000,000 population were calculated. The Joinpoint Regression Program was used to evaluate annual percentage changes (APCs) in mortality trends, with statistical significance set at *P*<.05. This study adhered to the STROBE (Strengthening the Reporting of Observational Studies in Epidemiology) guidelines for reporting.

**Results:**

Between 1999 and 2022, a total of 425,777 deaths were recorded from DM and pneumonia. The overall AAMR declined significantly (*P*=.001) from 98.73 in 1999 to 49.17 in 2016 (APC –4.68), and then surged to 97.66 by 2022 (APC 23.55). Men consistently experienced higher mortality than women throughout the study period. Male AAMR rose from 62.61 in 2016 to 127.05 in 2022 (APC 24.88), while female AAMR increased from 41.05 in 2017 to 75.25 in 2022 (APC 27.60). Race-based analysis demonstrated that American Indian or Alaska Native populations had the highest mortality rates among racial groups. Non-Hispanic White individuals exhibited a significant decline in AAMR (*P*=.002) from 89.76 in 1999 to 44.19 in 2017 (APC −4.58), followed by an increase to 83.11 by 2022 (APC 25.25). Adults aged 65 years or older bore the highest mortality burden, with rates declining steadily to 206.9 in 2017 (APC –5.15) before rising sharply to 371.3 in 2022 (APC 20.01). Nonmetropolitan areas consistently exhibited higher mortality than metropolitan areas, with particularly steep increases after 2018 (APC 64.42). Type-specific mortality revealed that type 1 DM AAMRs declined from 9.2 in 1999 to 1.4 in 2015 (APC –11.94) before rising again. By contrast, type 2 DM AAMRs surged drastically after 2017, peaking at 62.2 in 2020 (APC 58.74) before partially declining to 41.6 by 2022.

**Conclusions:**

DM is associated with an increased risk of mortality following pneumonia, particularly among men, older adults, and American Indian populations. Strengthening health care interventions and policies is essential to curb the rising mortality trend in these at-risk groups.

## Introduction

Approximately 10% of the US population has diabetes mellitus (DM) [1]. Patients with DM are more susceptible to complications from infectious diseases and are reported to have a 4.4-fold higher risk of bacteremia than those without DM [2].

Among individuals with DM, pneumonia is the most common infection managed in hospitals and the third most common infection treated in emergency departments in the United States [1]. Individuals with diabetes are more prone to pneumonia due to various factors, including hyperglycemia-induced immune dysregulation, declining lung function, and coexisting comorbidities such as cerebrovascular events, cardiovascular disease (CVD), and chronic kidney disease (CKD), all of which adversely impact outcomes in pneumonia [3-5]. However, population-level data on mortality trends for co-occurring DM and pneumonia, particularly across demographic strata, remain limited [6,7]. Therefore, the objective of this study was to evaluate the demographic and regional differences in mortality patterns among patients with both pneumonia and DM.

## Methods

### Screening of Data

The Centers for Disease Control and Prevention Wide-ranging Online Data for Epidemiologic Research (CDC WONDER) database was used for this research. The CDC WONDER database comprises death certificates from all 50 US states and the District of Columbia. Patients with DM were identified using the International Statistical Classification of Diseases and Related Health Problems, 10th Revision (ICD-10) codes E10-E14, whereas patients with pneumonia were identified with the ICD-10 codes J09-J18. These ICD-10 codes have also been used in previous studies [8-10]. We used the Multiple Cause-of-Death Public Use Record death certificates to select records in which both DM and pneumonia were listed as either contributing or underlying causes of death [11]. Adults in our study were defined as individuals aged 25 years or older, consistent with prior CDC WONDER studies that use this threshold to define adulthood in the context of chronic disease mortality trends [12,13]. While this excludes younger populations, deaths in these age groups are rare and less likely to reflect long-term cardiometabolic complications.

### Ethics Considerations

The data provided in the database are deidentified by the government; therefore, our study is exempt from institutional review board approval. The STROBE (Strengthening the Reporting of Observational Studies in Epidemiology) guidelines were followed for reporting.

### Data Extraction

Data on deaths due to coexisting DM and pneumonia, including population size and location, were extracted for the period between January 1999 and December 2022. Demographic variables, such as gender/sex, race/ethnicity, age groups, urban-rural classification, and census regions, were analyzed for the same period. Based on previous analyses of the CDC WONDER database, race/ethnicity was categorized as Hispanic or Latino; Non-Hispanic (NH) American Indians or Alaska Natives (ANs); NH Asian or Pacific Islanders; NH Black or African Americans; and NH White individuals. This classification is based on data reported on death certificates in accordance with the US Office of Budget and Management Guidelines [14]. Census regions were divided into Northeast, Midwest, West, and South, based on US Census Bureau definitions. The National Center for Health Statistics Urban-Rural Classification Scheme was used to categorize the population into 2 groups, based on the 2013 US Census: metropolitan (large metropolitan area [population ≥1 million] and medium/small metropolitan area [population 50,000‐999,999]) and nonmetropolitan (population <50,000). While the CDC WONDER database provides nationally representative data, it lacks individual-level clinical variables (eg, vital signs and treatment details), which are crucial for understanding the extent of disease.

### Data Analysis

The age-adjusted mortality rate (AAMR) per 1,000,000 people was calculated by standardizing DM- and pneumonia-related deaths to the US population in 2000 [15]. AAMRs for all demographic variables, including sex/gender, race/ethnicity, age groups, urbanization status, and census regions, were calculated, as the CDC WONDER database provides population sizes stratified by demographic and regional factors and age groups for each specific year [14].

The data for the urbanization group differed from all the other variables in that the AAMRs could only be calculated up to 2020. This is because standardized population data for 2021 and 2022 are not available in the CDC WONDER database. The database also provided 95% CIs for the AAMRs. Trends in AAMRs were analyzed using the Joinpoint Regression Program (version 5.0; National Cancer Institute), which calculates the annual percentage change (APC) [16]. This program uses log-linear regression models to detect temporal variations and identify significant changes in AAMRs over time. APCs with 95% CIs for AAMR were estimated at the identified line segments connecting the joinpoints, using the Monte Carlo permutation test. Based on the results of the 2-tailed *t* test, APCs were considered to have significantly increased or decreased only if the slope representing the change in mortality differed significantly from 0. The significance level was set at *P*<.05.

### Trend Analysis

Temporal trends in annual AAMRs from 1999 to 2022 were analyzed using the Joinpoint Regression Program (version 5.0; National Cancer Institute). Rates were log-transformed and modeled to identify inflection points in the trend. Joinpoint settings were as follows: minimum joinpoints=0 and maximum=4, with a minimum of 2 observations required before the first joinpoint, after the last joinpoint, and between joinpoints. Model selection used the weighted Bayesian information criterion (weighted BIC), with the permutation test seed set to 7160 and the empirical quantile seed set to 10,000 for reproducibility. For the overall data, the weighted BIC selected 2 joinpoints. Subgroup analyses by sex, age group, race/ethnicity, urban-rural classification, region, and diabetes type used identical settings; the weighted BIC also selected 2 joinpoints in each subgroup, facilitating consistent comparison of segment timing. APCs and 95% CIs were reported for each segment. Observed versus fitted rates were exported (Export.Data.txt); residuals on the log scale (Residual=ln[observed rate] − ln[fitted rate]) were computed. For the overall model, the Durbin-Watson statistic was 1.62, indicating mild positive autocorrelation. Basic visual inspection of residuals did not reveal extreme or systematic patterns. Formal residual autocorrelation tests and sensitivity analyses (eg, varying the minimum number of observations between joinpoints or the maximum number of joinpoints) were not performed for subgroups due to resource constraints. Nonetheless, we considered the assumption of independence to be approximately reasonable, while acknowledging this as a limitation.

### Ethical Considerations

This study utilized data from the CDC WONDER database, a publicly available dataset [17]. Therefore, institutional review board approval and informed consent are not applicable.

## Results

### Annual Trends for Overall Diabetes- and Pneumonia-Related Mortality

**Figure 1.**
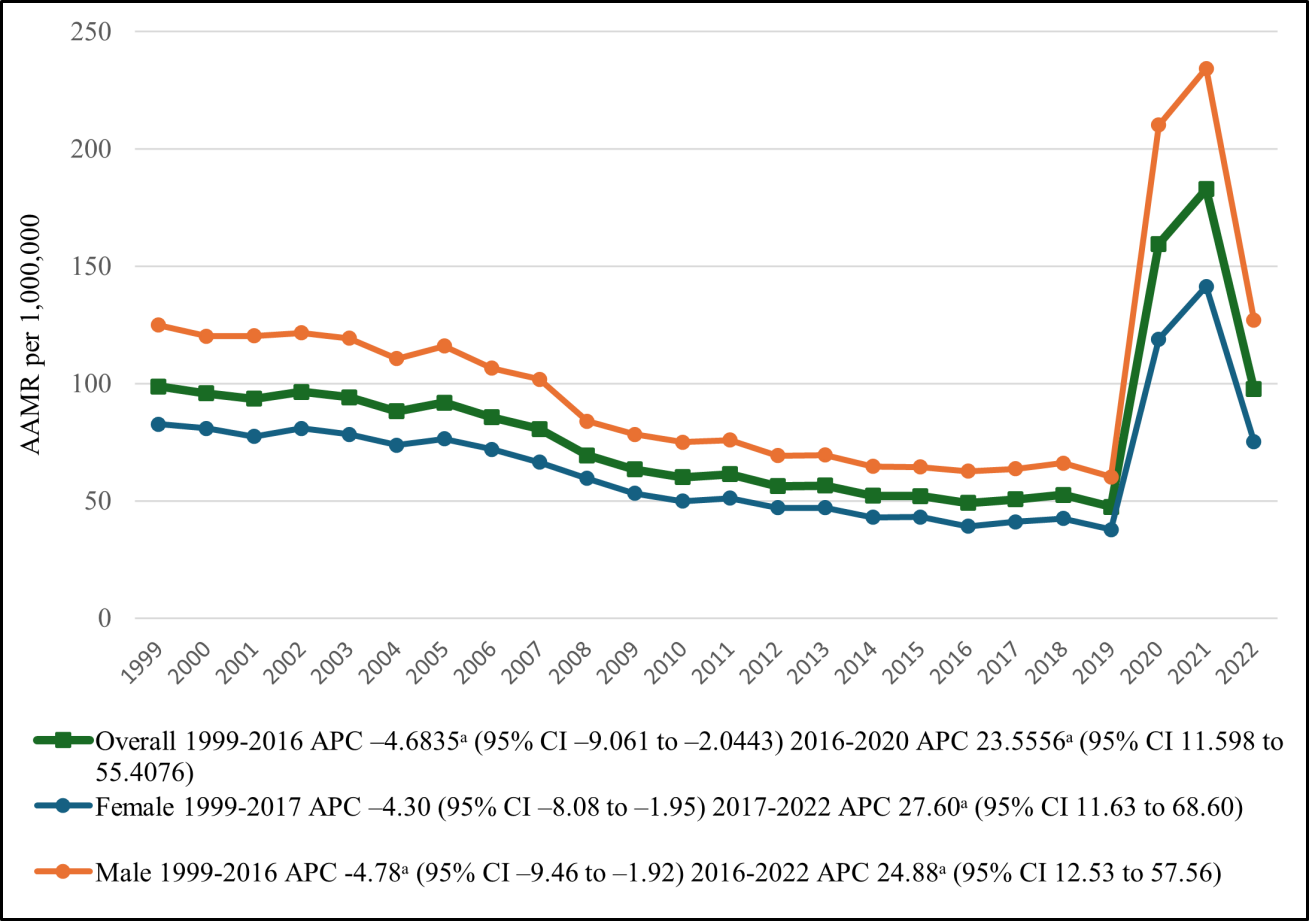
Overall and sex-stratified diabetes mellitus– and pneumonia-related age-adjusted mortality rates (AAMRs) per 1,000,000 in the United States, 1999-2022. ^a^The APC is significantly different from zero at α=.05. APC: annual percentage change.

A total of 425,777 deaths related to DM and pneumonia occurred between 1999 and 2022. The overall AAMR for DM and pneumonia among individuals aged 25 years and above was 98.73 at the start of the study period in 1999, which decreased to 49.17 by 2016 (APC −4.68, 95% CI −9.06 to −2.04). From 2016 onward, there was a significant increase in the AAMR (*P*=.001), reaching 97.66 by 2022 (APC 23.55, 95% CI 11.59-55.40; Table 1, Figure 1).

**Table 1.** Annual percent change in age-adjusted mortality rates per 1,000,000 for diabetes mellitus– and pneumonia-related deaths among older adults in the United States, 1999-2022.

Year interval	Annual percent change (95% CI)
Overall	
1999‐2016	−4.68^a^ (−9.06 to −2.04)
2016‐2022	23.56^a^ (11.60 to 55.41)
Men	
1999‐2016	−4.78^a^ (−9.47 to −1.93)
2016‐2022	24.89^a^ (12.54 to 57.56)
Women	
1999‐2017	−4.30^a^ (−8.08 to −1.96)
2017‐2022	27.61^a^ (11.63 to 68.61)
Young age	
1999‐2017	0.92 (−8.91 to 5.42)
2017‐2022	40.34^a^ (15.99 to 124.26)
Middle age	
1999‐2016	−2.34 (−10.49 to 2.44)
2016‐2022	35.78^a^ (17.83 to 93.68)
Old age	
1999‐2016	−5.16^a^ (−8.76 to −2.95)
2016‐2022	20.01^a^ (9.84 to 46.03)
NH^b^ White	
1999‐2017	−4.59^a^ (−7.22 to −2.78)
2017‐2022	25.25^a^ (12.68 to 53.96)
NH American Indian or Alaska Native	
1999‐2016	−3.18 (−11.92 to 1.59)
2016‐2022	29.34^a^ (13.82 to 72.43)
NH Black or African American	
1999‐2016	−4.86^a^ (−11.21 to −1.50)
2016‐2022	25.28^a^ (10.59 to 64.76)
Hispanic or Latino	
1999‐2016	−3.75 (−18.13 to 2.64)
2016‐2022	32.28^a^ (11.98 to 96.77)
NH Asian or Pacific Islander	
1999‐2016	−5.17^a^ (−11.18 to −1.49)
2016‐2022	20.37^a^ (8.38 to 52.55)
Nonmetropolitan areas	
1999‐2018	−3.87^a^ (−5.35 to −2.74)
2018‐2020	64.43^a^ (32.82 to 83.88)
Metropolitan area	
1999‐2018	−4.64^a^ (−6.50 to −3.28)
2018‐2020	85.94^a^ (48.88 to 110.37)

a Indicates that the APC is significantly different from zero at α=.05.

b NH: non-Hispanic.

### Annual Trends Stratified by Sex

Of the 425,777 deaths, 224,323 (52.69%) were men, and 201,454 (47.31%) were women. Overall, men consistently had higher AAMRs than women throughout the study period. Among men, the AAMR declined from 1999 to 2016 (APC –4.78, 95% CI –9.46 to –1.92), followed by a sharp increase from 62.61 in 2016 to 127.05 in 2022 (APC 24.88, 95% CI 12.53-57.56). By contrast, women’s AAMR declined from 1999 to 2017 (APC −4.30, 95% CI−8.08 to −1.95), then rose markedly from 41.05 in 2017 to 75.25 in 2022 (APC 27.60, 95% CI 11.63-68.60; Tables1  2, Figure 1).

**Table 2.** Overall and sex‐stratified diabetes mellitus– and pneumonia-related age-adjusted mortality rates per 1,000,000 in the United States, 1999-2022.

Year	Men, age-adjusted rate (95% CI)	Women, age-adjusted rate (95% CI)	Overall, age-adjusted rate (95% CI)
1999	124.94 (122.22‐127.67)	82.67 (80.95‐84.39)	98.73 (97.27‐100.20)
2000	120.21 (117.56‐122.86)	80.87 (79.18‐82.56)	95.82 (94.38‐97.26)
2001	120.32 (117.70‐122.95)	77.44 (75.79‐79.09)	93.53 (92.12‐94.94)
2002	121.61 (119.00‐124.22)	80.96 (79.28‐82.63)	96.51 (95.09‐97.93)
2003	119.27 (116.72‐121.82)	78.32 (76.68‐79.95)	94.13 (92.73‐95.52)
2004	110.65 (108.22‐113.07)	73.80 (72.22‐75.38)	88.18 (86.84‐89.51)
2005	115.99 (113.54‐118.44)	76.39 (74.79‐77.99)	91.90 (90.54‐93.25)
2006	106.52 (104.21‐108.84)	72.03 (70.48‐73.57)	85.61 (84.31‐86.90)
2007	101.81 (99.58‐104.04)	66.54 (65.06‐68.01)	80.63 (79.38‐81.87)
2008	83.98 (81.98‐85.98)	59.59 (58.21‐60.97)	69.43 (68.29‐70.58)
2009	78.34 (76.43‐80.24)	53.22 (51.91‐54.52)	63.39 (62.31‐64.48)
2010	75.05 (73.20‐76.90)	49.86 (48.61‐51.11)	60.15 (59.10‐61.20)
2011	75.95 (74.12‐77.77)	51.17 (49.92‐52.42)	61.39 (60.34‐62.43)
2012	69.24 (67.52‐70.96)	47.08 (45.89‐48.28)	56.22 (55.23‐57.21)
2013	69.54 (67.84‐71.23)	47.04 (45.85‐48.22)	56.54 (55.55‐57.52)
2014	64.72 (63.11‐66.33)	43.00 (41.88‐44.13)	52.16 (51.23‐53.10)
2015	64.43 (62.84‐66.02)	43.10 (41.99‐44.21)	52.09 (51.16‐53.01)
2016	62.61 (61.07‐64.15)	39.20 (38.15‐40.26)	49.17 (48.28‐50.06)
2017	63.63 (62.09‐65.17)	41.05 (39.98‐42.12)	50.61 (49.72‐51.50)
2018	66.07 (64.53‐67.62)	42.47 (41.39‐43.55)	52.59 (51.69‐53.49)
2019	60.20 (58.75‐61.65)	37.82 (36.81‐38.83)	47.53 (46.68‐48.37)
2020	210.24 (207.58‐212.90)	119.00 (117.20‐120.79)	159.52 (157.98‐161.06)
2021	234.26 (231.42‐237.10)	75.25 (73.83‐76.66)	182.84 (181.17‐184.52)
2022	127.05 (124.98‐129.12)	83.69 (83.01‐84.38)	97.66 (96.47‐98.86)
Total	99.43 (99.02‐99.84)	99.43 (99.02‐99.84)	80.65 (80.00‐81.30)

### Annual Trends Stratified by Race

**Figure 2.**
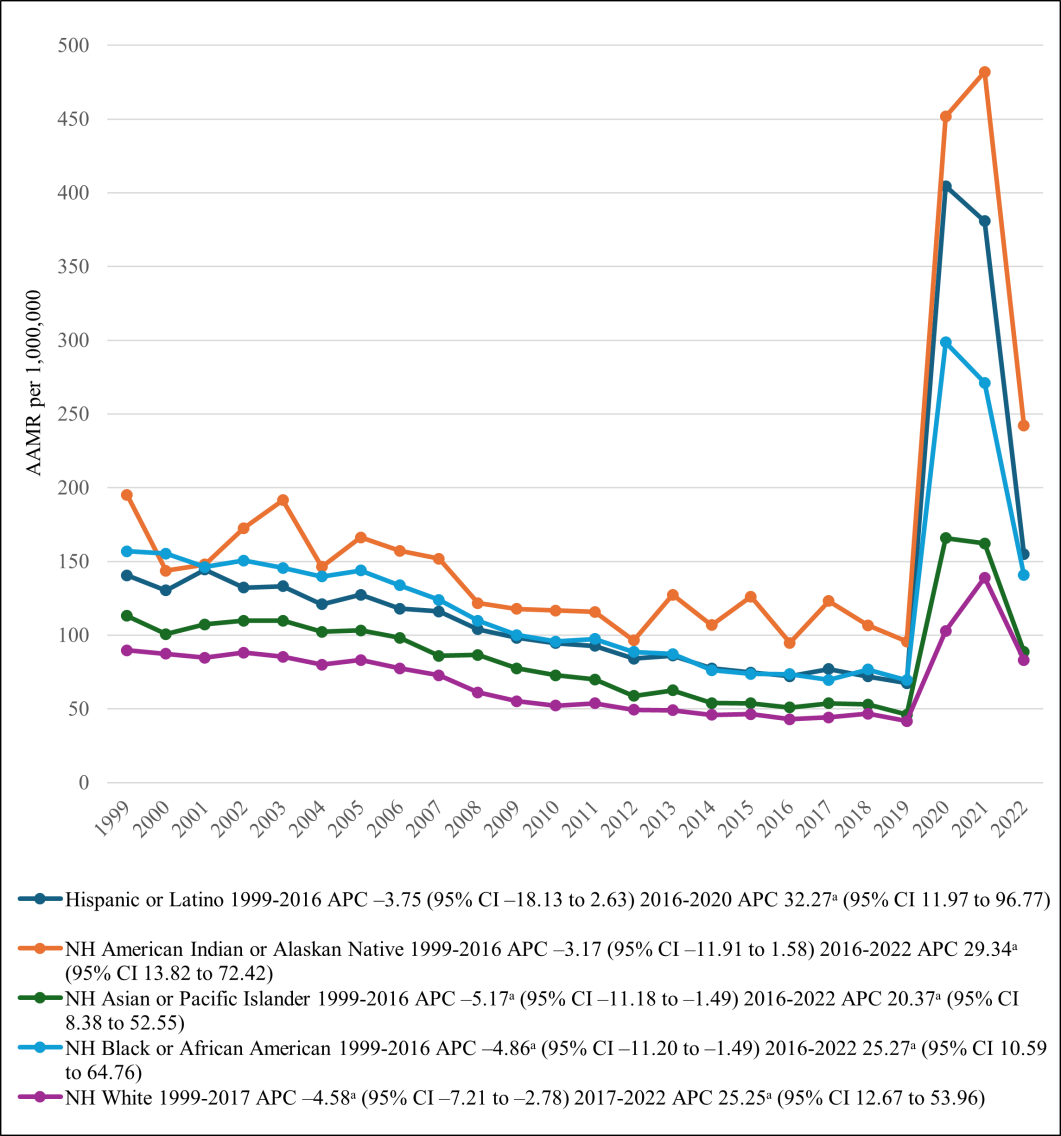
Diabetes mellitus– and pneumonia-related age-adjusted mortality rates (AAMRs) per 1,000,000, stratified by race in the United States, 1999-2022. ^a^The APC is significantly different from zero at *α*=.05. APC: annual percentage change; NH: non-Hispanic.

Racial analysis revealed that the NH White population had the highest burden of deaths (287,507/425,777, 67.53%), followed by NH Black or African American (60,507/425,777, 14.21%), Hispanic or Latino (56,115/425,777, 13.18%), and NH Asian or Pacific Islander individuals (16744/425,777, 3.93%). NH AI or AN individuals had the lowest number of reported deaths (4904/425,777, 1.15%). After stratification by race/ethnicity, AAMRs were the highest among AI or AN individuals, followed by Hispanic or Latino, NH Black or African American, NH Asian or Pacific Islander, and NH White individuals. The AAMR of both NH Black or African American and NH Asian or Pacific Islander individuals decreased significantly (*P*=.04) from 1999 to 2016, followed by a significant steep increase (*P*=.04) through 2022. Hispanic or Latino and NH AI individuals differed in that their AAMRs did not show a significant decrease (*P*=.06) from 1999 to 2016; however, from 2016 onward, there was a significant increase (*P*=.001) until 2022. NH White individuals differed from all other racial groups in that their AAMR decreased significantly (*P*=.002) from 89.76 in 1999 to 44.19 in 2017 (APC –4.58, 95% CI −7.21 to −2.78), followed by a significant increase (*P*=.001), with the AAMR reaching 83.11 by 2022 (APC 25.25, 95% CI 12.67-53.96; Tables1  3, Figure 2).

**Table 3.** Diabetes mellitus– and pneumonia–related age-adjusted mortality rates per 1,000,000.

Year	NH Asian or Pacific Islander, age-adjusted rate (95% CI)	NH^a^ American Indian or Alaska Native, age-adjusted rate (95% CI)	NH Black or African American, age-adjusted rate (95% CI)	NH White, age-adjusted rate (95% CI)	Hispanic or Latino, age-adjusted rate (95% CI)
1999	113.1 (101.59‐124.62)	195.13 (159.88‐230.37)	156.91 (150.42‐163.41)	89.76 (88.24‐91.28)	140.62 (132.13‐149.11)
2000	100.77 (90.30‐111.24)	143.76 (114.68‐172.85)	155.40 (148.96‐161.84)	87.43 (85.93‐88.92)	130.35 (122.40‐138.30)
2001	107.30 (96.94‐117.65)	148.00 (119.17‐176.82)	146.15 (139.95‐152.35)	84.66 (83.20‐86.12)	144.52 (136.35‐152.69)
2002	109.89 (99.79‐120.00)	172.57 (141.61‐203.53)	150.69 (144.42‐156.96)	88.13 (86.65‐89.62)	132.26 (124.66‐139.86)
2003	109.78 (100.00‐119.57)	191.5 (159.06‐223.95)	145.56 (139.43‐151.68)	85.43 (83.98‐86.88)	133.28 (125.88‐140.69)
2004	102.32 (93.13‐111.52)	146.36 (119.32‐173.39)	139.77 (133.83‐145.72)	80.00 (78.60‐81.40)	120.92 (114.04‐127.80)
2005	103.23 (94.38‐112.09)	166.35 (136.73‐195.98)	143.93 (138.00‐149.86)	83.14 (81.72‐84.56)	127.45 (120.62‐134.28)
2006	98.25 (89.87‐106.64)	157.21 (129.67‐184.75)	133.91 (128.23‐139.58)	77.48 (76.12‐78.84)	118.05 (111.62‐124.48)
2007	86.04 (78.42‐93.67)	151.92 (125.28‐178.55)	123.99 (118.60‐129.39)	72.87 (71.56‐74.18)	116.12 (109.91‐122.33)
2008	86.60 (79.18‐94.01)	121.77 (99.20‐144.34)	109.82 (104.82‐114.82)	61.26 (60.06‐62.45)	103.98 (98.28‐109.68)
2009	77.51 (70.76‐84.27)	117.82 (95.53‐140.12)	100.07 (95.36‐104.78)	55.31 (54.18‐56.45)	98.18 (92.89‐103.48)
2010	72.76 (66.35‐79.17)	116.68 (94.89‐138.48)	95.65 (91.08‐100.22)	52.31 (51.22‐53.40)	94.56 (89.39‐99.72)
2011	69.99 (63.99‐76.00)	115.75 (94.65‐136.85)	97.48 (92.94‐102.01)	53.76 (52.67‐54.86)	92.69 (87.78‐97.59)
2012	58.84 (53.49‐64.19)	96.50 (77.69‐115.31)	88.72 (84.48‐92.95)	49.47 (48.42‐50.52)	84.14 (79.62‐88.67)
2013	62.55 (57.25‐67.85)	127.44 (106.49‐148.39)	87.25 (83.13‐91.38)	49.14 (48.10‐50.17)	86.18 (81.73‐90.62)
2014	53.90 (49.18‐58.62)	106.82 (88.30‐125.34)	76.19 (72.41‐79.97)	45.98 (44.98‐46.98)	77.53 (73.45‐81.60)
2015	53.82 (49.26‐58.39)	126.13 (106.66‐145.61)	73.77 (70.11‐77.43)	46.40 (45.40‐47.40)	74.72 (70.83‐78.62)
2016	50.94 (46.63‐55.25)	94.76 (77.77‐111.75)	73.60 (70.00‐77.20)	42.98 (42.03‐43.93)	72.22 (68.49‐75.96)
2017	53.83 (49.53‐58.13)	123.20 (104.90‐141.50)	69.60 (66.17‐73.03)	44.19 (43.23‐45.15)	77.01 (73.28‐80.74)
2018	53.12 (48.98‐57.25)	106.65 (89.98‐123.32)	76.74 (73.19‐80.29)	46.68 (45.70‐47.67)	72.08 (68.55‐75.62)
2019	46.20 (42.46‐49.95)	95.65 (80.41‐110.89)	69.58 (66.25‐72.91)	41.71 (40.79‐42.63)	67.54 (64.20‐70.88)
2020	165.78 (158.90‐172.67)	451.73 (419.44‐484.02)	298.61 (291.87‐305.35)	102.85 (101.41‐104.28)	404.48 (396.66‐412.31)
2021	85.50 (80.60‐90.41)	482.03 (447.47‐516.60)	271.20 (264.75‐277.64)	138.97 (137.24‐140.69)	380.84 (373.36‐388.32)
2022	88.75 (83.82‐93.68)	242.20 (218.02‐266.38)	140.80 (136.19‐145.41)	83.11 (81.81‐84.40)	155.03 (150.24‐159.82)
Total	91.01 (89.48‐92.54)	168.31 (163.10‐173.52)	130.47 (129.39‐131.55)	67.42 (67.15‐67.69)	130.53 (129.26‐131.80)

a NH: non-Hispanic.

### Annual Trends Stratified by Age Groups

**Figure 3.**
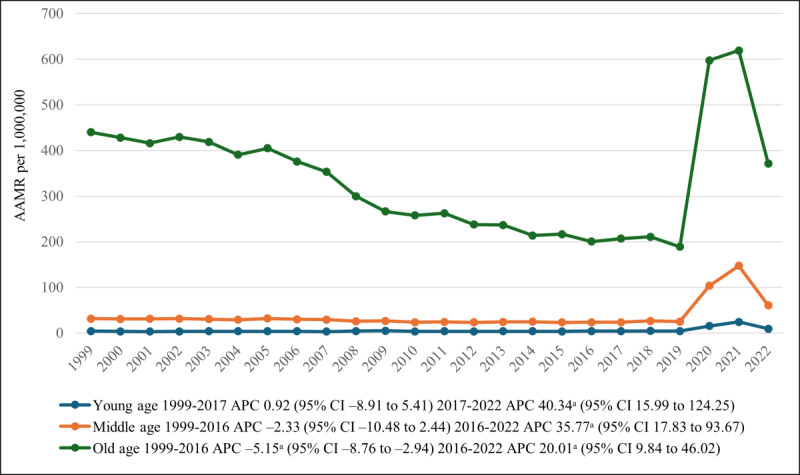
Diabetes mellitus– and pneumonia-related age-adjusted mortality rates (AAMRs) per 1,000,000, stratified by age group in the United States, 1999-2022. ^a^The APC is significantly different from zero at α=.05. APC: annual percentage change.

The older adult group (65‐85+ years) accounted for a whopping 341,295 deaths (constituting 80.16% of all mortality; N=425,777), distantly followed by the middle-aged (45‐64 years) and young adult groups (25‐44 years), with 73,817 (17.34%) and 10,665 (2.50%) deaths, respectively. Overall, the older adult group (65‐85+ years) had the highest AAMR, followed by the middle-aged group (45‐64 years) and the young adult group (25‐44 years). Starting in 1999, both young adult and middle-aged group AAMRs remained relatively stable until 2016, followed by a significant and dramatic increase (*P*=.001) through 2022. Older adults differed from the other 2 groups in that their AAMR steadily decreased from 440.9 in 1999 to 206.9 in 2017 (APC −5.15, 95% CI −8.76 to −2.94). From 2017 to 2022, the AAMR significantly increased (*P*=.001), reaching 371.3 (APC 20.01, 95% CI 9.84-46.02; Tables1  4, Figure 3).

**Table 4.** Diabetes mellitus– and pneumonia-related age-adjusted mortality rates per 1,000,000, stratified by age groups in the United States, 1999-2022.

Year	Young adults (25‐44 years), age-adjusted rate (95% CI)	Middle aged (45‐64 years), age-adjusted rate (95% CI)	Older adults (65‐85+ years), age-adjusted rate (95% CI)
1999	4.13 (3.69‐4.56)	31.50 (30.09‐32.92)	440.09 (433.08‐447.10)
2000	3.45 (3.06‐3.85)	30.74 (29.35‐32.12)	428.12 (421.24‐434.99)
2001	3.27 (2.88‐3.66)	31.16 (29.79‐32.52)	416.08 (409.35‐422.81)
2002	3.66 (3.25‐4.08)	31.64 (30.30‐32.99)	429.58 (422.78‐436.38)
2003	3.82 (3.39‐4.24)	30.52 (29.23‐31.82)	418.96 (412.29‐425.63)
2004	3.98 (3.55‐4.42)	29.07 (27.83‐30.31)	390.69 (384.28‐397.10)
2005	3.93 (3.49‐4.36)	31.95 (30.67‐33.23)	404.79 (398.32‐411.26)
2006	3.88 (3.45‐4.31)	30.06 (28.85‐31.28)	376.03 (369.85‐382.22)
2007	3.31 (2.91‐3.71)	29.37 (28.18‐30.56)	353.14 (347.19‐359.08)
2008	4.13 (3.68‐4.58)	26.02 (24.91‐27.13)	299.79 (294.37‐305.21)
2009	4.89 (4.40‐5.38)	26.45 (25.34‐27.55)	266.35 (261.28‐271.42)
2010	3.63 (3.20‐4.05)	23.55 (22.52‐24.58)	257.81 (252.86‐262.76)
2011	3.85 (3.41‐4.28)	24.24 (23.20‐25.27)	262.43 (257.50‐267.36)
2012	3.75 (3.32‐4.18)	23.36 (22.34‐24.38)	237.75 (233.11‐242.39)
2013	3.99 (3.55‐4.43)	24.45 (23.42‐25.48)	236.90 (232.33‐241.47)
2014	4.08 (3.63‐4.53)	24.81 (23.77‐25.85)	213.68 (209.38‐217.97)
2015	3.65 (3.23‐4.08)	23.39 (22.39‐24.40)	216.77 (212.50‐221.04)
2016	4.18 (3.73‐4.63)	23.51 (22.51‐24.52)	200.37 (196.31‐204.43)
2017	4.27 (3.82‐4.73)	23.86 (22.85‐24.88)	206.93 (202.87‐211.00)
2018	4.80 (4.32‐5.28)	26.74 (25.65‐27.82)	210.76 (206.72‐214.81)
2019	4.21 (3.76‐4.66)	25.15 (24.09‐26.21)	189.05 (185.28‐192.82)
2020	15.31 (14.46‐16.16)	103.79 (101.64‐105.94)	597.69 (591.07‐604.30)
2021	24.31 (23.23‐25.38)	147.52 (144.93‐150.11)	618.97 (612.14‐625.79)
2022	9.16 (8.51‐9.82)	60.75 (59.09‐62.40)	371.34 (366.18‐376.49)
Total	5.53 (5.41‐5.65)	34.09 (33.82‐34.36)	330.07 (329.01‐331.13)

### Annual Trends Stratified by Geographic Region

**Figure 4.**
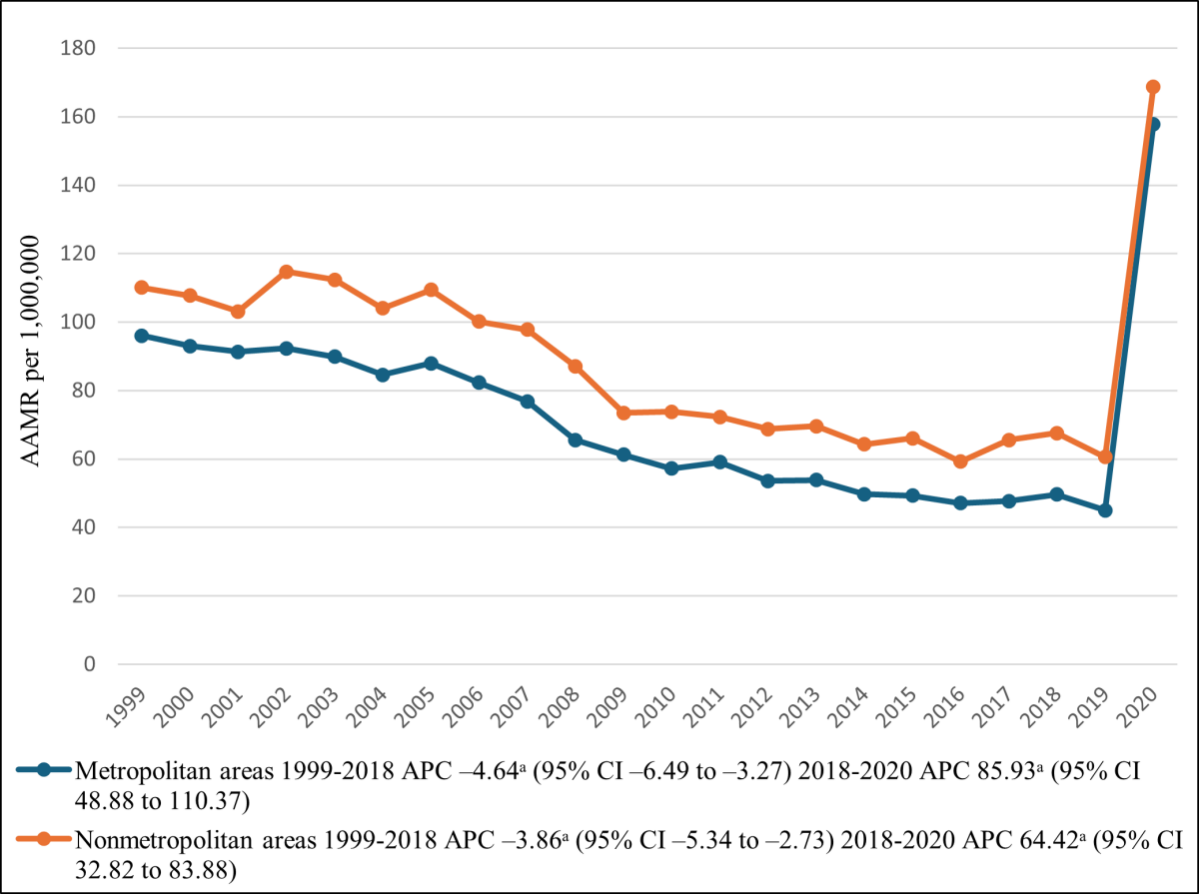
Diabetes mellitus– and pneumonia-related age-adjusted mortality rates (AAMRs) per 1,000,000, stratified by urbanization group in the United States, 1999-2020. ^a^The APC is significantly different from zero at α=.05. APC: annual percentage change.

As data on urbanization status are available only up to 2020, a total of 351,840 deaths could be recorded, with 278,378 (79.12%) deaths occurring in metropolitan areas and 73,462 (20.88%) in nonmetropolitan areas. Overall, nonmetropolitan areas had higher AAMRs than metropolitan areas. The AAMR in metropolitan areas decreased from 1999 to 2018 (APC −4.64, 95% CI−6.49 to −3.27), followed by a significantly steeper increase (*P*=.002) through 2020 (APC 85.93, 95% CI 48.88 to −110.37). The AAMR in nonmetropolitan areas followed a similar trend, decreasing from 110 in 1999 to 67.61 in 2018 (APC −3.86, 95% CI −5.34 to −2.73). By 2020, the AAMR had significantly increased (*P*=.002) to 168.76 (APC 64.42, 95% CI 32.82-83.88; Tables1  5, Figure 4).

**Figure 5.**
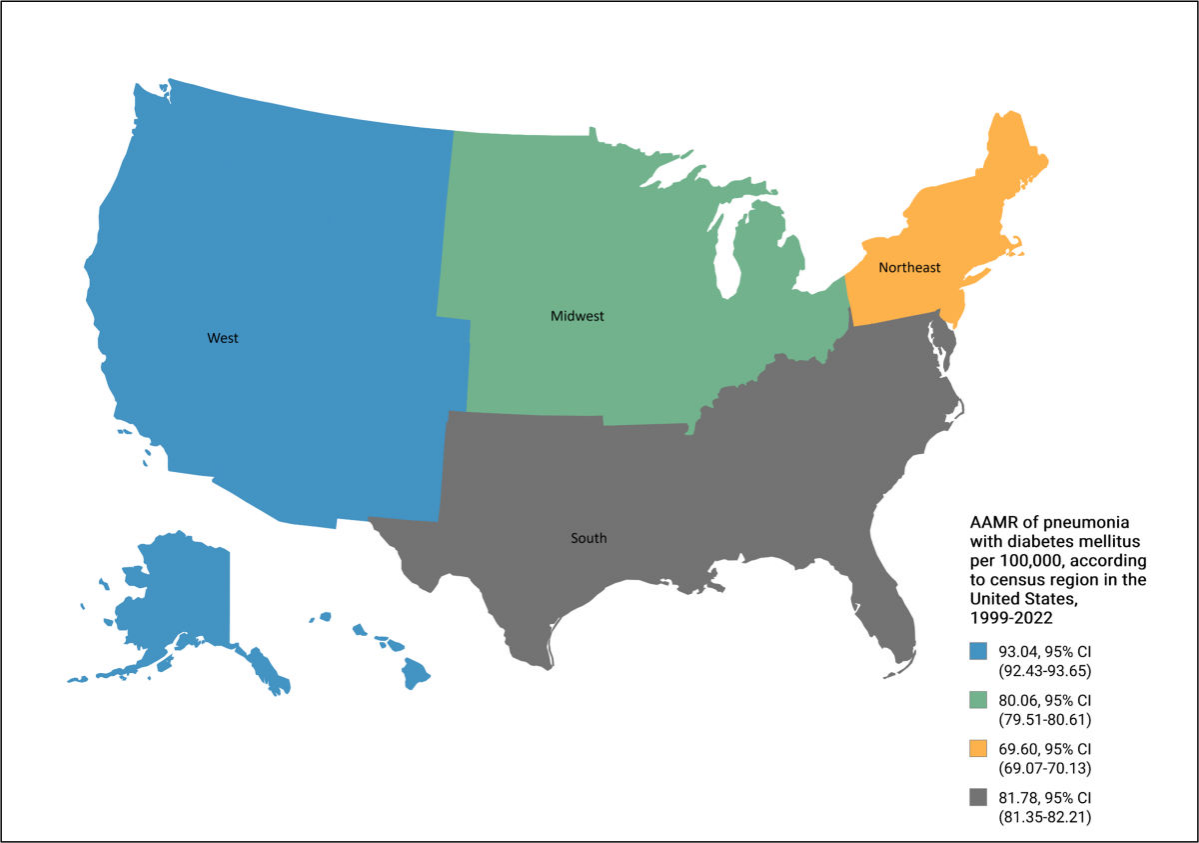
Diabetes mellitus– and pneumonia-related age-adjusted mortality rates (AAMRs) per 1,000,000, stratified by census regions in the United States, 1999-2022.

The South region bore the greatest burden of deaths, with 159,187 out of 425,777 (37.39%) fatalities, followed by the West (104,971 deaths, 24.65%), Midwest (92,161 deaths, 21.65%), and Northeast (69,458 deaths, 16.31%) regions. Overall, the AAMR was highest in the West region, followed by the South, Midwest, and Northeast regions. The Northeast and South regions showed a significant decrease in AAMR (*P*=.002) from 1999 to 2016, followed by a significant increase (*P*=.001) through 2022. By contrast, the West and Midwest regions experienced a decrease in AAMR from 1999 to 2017, after which, similar to the other 2 regions, AAMRs increased through 2022 (Tables1  6, Figure 5).

**Table 5.** Diabetes mellitus– and pneumonia–related age-adjusted mortality rates per 1,000,000, stratified by urban-rural classification in the United States, 1999-2022.

Year	Metropolitan, age-adjusted rate (95% CI)	Nonmetropolitan, age-adjusted rate (95% CI)
1999	96.03 (94.42‐97.64)	110.06 (106.51‐113.60)
2000	92.97 (91.39‐94.54)	107.76 (104.27‐111.25)
2001	91.32 (89.77‐92.86)	103.06 (99.66‐106.47)
2002	92.31 (90.76‐93.85)	114.75 (111.17‐118.33)
2003	89.87 (88.36‐91.37)	112.40 (108.87‐115.93)
2004	84.59 (83.14‐86.04)	103.99 (100.61‐107.38)
2005	87.91 (86.45‐89.38)	109.42 (105.97‐112.88)
2006	82.32 (80.92‐83.73)	100.17 (96.89‐103.45)
2007	76.80 (75.45‐78.14)	97.81 (94.58‐101.03)
2008	65.55 (64.32‐66.78)	87.08 (84.05‐90.11)
2009	61.24 (60.06‐62.41)	73.47 (70.68‐76.25)
2010	57.18 (56.05‐58.30)	73.81 (71.04‐76.58)
2011	59.06 (57.93‐60.19)	72.27 (69.55‐74.99)
2012	53.62 (52.56‐54.69)	68.73 (66.10‐71.37)
2013	53.85 (52.79‐54.90)	69.63 (66.99‐72.27)
2014	49.71 (48.71‐50.71)	64.27 (61.74‐66.80)
2015	49.30 (48.31‐50.29)	66.08 (63.53‐68.62)
2016	47.14 (46.19‐48.09)	59.28 (56.88‐61.69)
2017	47.69 (46.75‐48.64)	65.58 (63.06‐68.10)
2018	49.67 (48.71‐50.62)	67.61 (65.06‐70.15)
2019	45.04 (44.14‐45.94)	60.55 (58.17‐62.94)
2020	157.81 (156.14‐159.48)	168.76 (164.77‐172.76)
Total	72.91 (72.64‐73.18)	89.34 (88.67‐90.01)

**Table 6.** Diabetes mellitus– and pneumonia-related age-adjusted mortality rates per 1,000,000, stratified by census region in the United States, 1999 to 2022.

Year	Northeast, age-adjusted rate (95% CI)	Midwest, age-adjusted rate (95% CI)	South, age-adjusted rate (95% CI)	West, age-adjusted rate (95% CI)
1999	90.91 (87.86‐93.96)	102.43 (99.38‐105.47)	92.78 (90.37‐95.19)	112.87 (109.33‐116.41)
2000	91.78 (88.73‐94.83)	94.04 (91.13‐96.94)	92.16 (89.78‐94.54)	108.30 (104.86‐111.73)
2001	85.79 (82.86‐88.71)	90.63 (87.79‐93.46)	90.67 (88.33‐93.02)	109.68 (106.27‐113.09)
2002	88.35 (85.40‐91.31)	96.13 (93.22‐99.04)	94.49 (92.11‐96.87)	108.47 (105.11‐111.82)
2003	84.25 (81.38‐87.13)	94.12 (91.26‐96.98)	90.91 (88.59‐93.22)	109.31 (105.98‐112.63)
2004	78.26 (75.50‐81.02)	90.11 (87.32‐92.90)	84.41 (82.21‐86.62)	102.05 (98.86‐105.24)
2005	79.82 (77.05‐82.59)	95.19 (92.34‐98.04)	87.53 (85.31‐89.76)	106.69 (103.48‐109.90)
2006	74.15 (71.50‐76.81)	87.82 (85.10‐90.55)	81.50 (79.38‐83.62)	100.67 (97.58‐103.76)
2007	68.48 (65.93‐71.03)	83.68 (81.05‐86.31)	78.02 (75.97‐80.08)	93.13 (90.20‐96.06)
2008	58.36 (56.03‐60.70)	71.57 (69.15‐73.99)	66.23 (64.36‐68.11)	82.67 (79.94‐85.40)
2009	52.10 (49.90‐54.30)	64.44 (62.16‐66.73)	61.98 (60.19‐63.77)	74.69 (72.13‐77.25)
2010	50.14 (48.00‐52.29)	58.43 (56.26‐60.59)	60.65 (58.89‐62.40)	69.96 (67.51‐72.41)
2011	53.66 (51.46‐55.86)	62.75 (60.52‐64.98)	57.56 (55.87‐59.24)	72.96 (70.49‐75.42)
2012	47.99 (45.92‐50.05)	55.36 (53.29‐57.43)	56.00 (54.36‐57.64)	64.31 (62.02‐66.59)
2013	48.51 (46.44‐50.57)	54.35 (52.31‐56.40)	56.96 (55.33‐58.59)	64.72 (62.47‐66.98)
2014	46.46 (44.44‐48.48)	50.81 (48.84‐52.77)	53.37 (51.81‐54.92)	55.97 (53.90‐58.04)
2015	46.78 (44.76‐48.79)	50.74 (48.79‐52.68)	52.42 (50.90‐53.94)	57.05 (54.99‐59.11)
2016	41.72 (39.83‐43.61)	46.14 (44.30‐47.99)	50.89 (49.41‐52.38)	55.31 (53.30‐57.31)
2017	41.19 (39.34‐43.04)	46.46 (44.63‐48.30)	52.08 (50.60‐53.56)	59.73 (57.68‐61.78)
2018	43.97 (42.06‐45.87)	49.82 (47.93‐51.71)	54.83 (53.32‐56.33)	58.20 (56.20‐60.19)
2019	41.30 (39.47‐43.13)	42.20 (40.48‐43.92)	50.40 (48.97‐51.82)	52.57 (50.69‐54.45)
2020	134.73 (131.42‐138.04)	146.56 (143.36‐149.76)	172.03 (169.44‐174.62)	169.59 (166.25‐172.92)
2021	96.86 (94.04‐99.69)	149.58 (146.27‐152.88)	215.70 (212.74‐218.66)	228.36 (224.42‐232.31)
2022	65.95 (63.66‐68.23)	88.47 (85.98‐90.96)	109.85 (107.80‐111.91)	111.59 (108.89‐114.29)
Total	69.60 (69.07‐70.13)	80.06 (79.51‐80.61)	81.78 (81.35‐82.21)	93.04 (92.43‐93.65)

### Type 1 Diabetes Mellitus–, Type 2 Diabetes Mellitus–, and Pneumonia-Related Mortality

**Figure 6.**
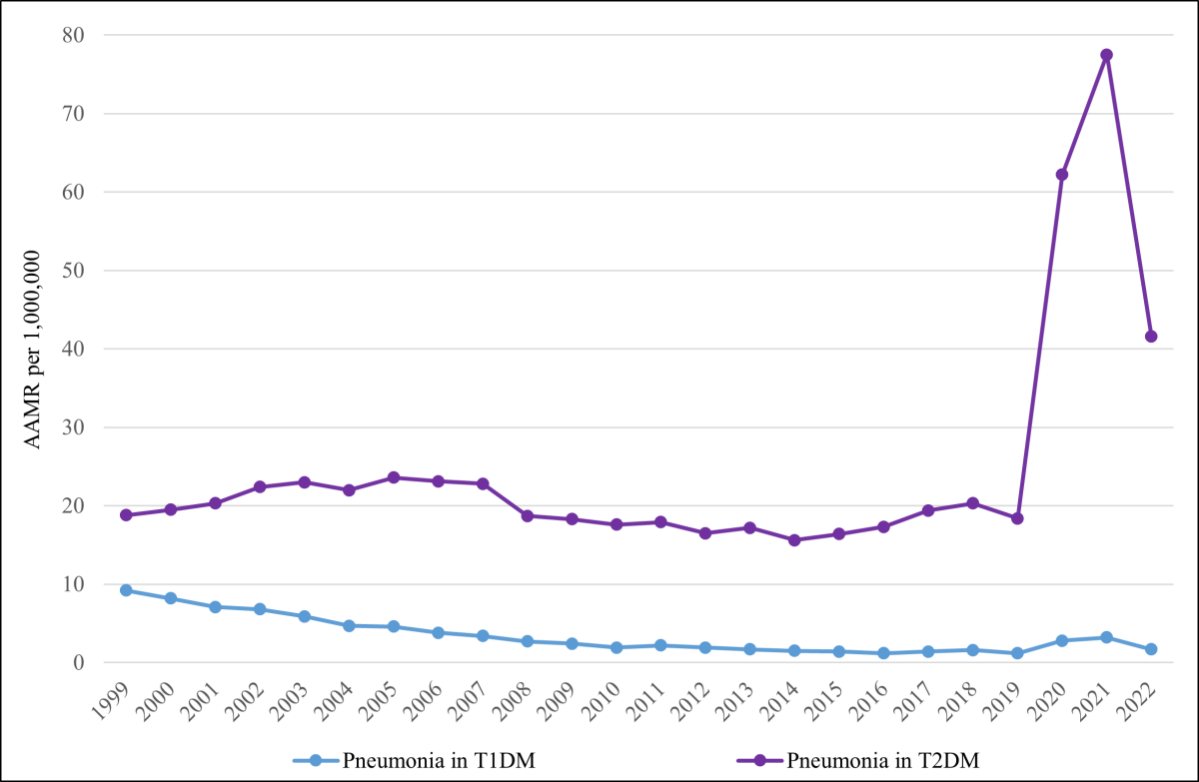
Diabetes mellitus– and pneumonia-related age-adjusted mortality rates (AAMRs) per 1,000,000 in the United States, 1999-2022, stratified by type of diabetes mellitus. T1DM: type 1 diabetes mellitus; T2DM: type 2 diabetes mellitus.

Based on the type of DM, diabetes- and pneumonia-related mortality exhibited a fascinating trend. The AAMR for type 1 DM and pneumonia-related mortality was 9.2 in 1999, dropping significantly (*P*=.04) to 1.4 by 2015 (APC −11.94, 95% CI −13.67 to −10.79). After 2015, the AAMR began increasing, reaching 3.2 by 2021; however, it dropped again to 1.7 in 2022. The overall trend from 2015 to 2022 was increasing in nature (APC 14.23, 95% CI 7.35-32.51). In terms of type 2 DM and pneumonia-related mortality, the AAMR remained relatively stable, from 18.8 in 1999 to 19.4 in 2017 (APC −2.09, 95% CI −6.27 to 2.42). From 2017, there was a drastic increase, and the AAMR reached a staggering 62.2 by 2020 (APC 58.74, 95% CI 10.56-81.43). After 2020, the AAMR began to decline, reaching 41.6, still higher than the previous baseline (APC −8.93, 95% CI −29.36 to 28.61; Figure 6).

## Discussion

### Principal Findings

In this 2-decade analysis, we showed an overall decrease in mortality due to DM and pneumonia, which aligns with previous literature [18]. This decline can be attributed to factors such as better glycemic control in patients with diabetes, thereby preventing infections; improved management of fatal cardiovascular outcomes in such patients; and enhanced treatment protocols for pneumonia, including the use of antibiotics and vaccines [18-20]. Furthermore, all demographic and regional trends revealed significant differences in mortality rates. Men, AIs/ANs, individuals aged 65‐86+ years, residents of nonmetropolitan areas, and those in the western region had higher mortality rates. In addition, a significant increase in mortality was observed during the COVID-19 pandemic.

### Gender Disparities

In our study, men were shown to have a higher mortality rate than women because of DM and pneumonia. This disparity can be attributed to sex differences in the prevalence of comorbidities such as CVD, smoking, and DM. Males are more inclined to engage in unfavorable lifestyle habits (eg, alcohol consumption, drug abuse, and smoking), which increases the risk for comorbidities such as hypertension, chronic obstructive pulmonary disease (COPD), DM, CKD, and CVD [20]. The increased frequency of DM in men is consistent with the literature and is primarily influenced by factors such as poor dietary practices, smoking, obesity, and alcoholism [21,22]. As these factors are more commonly observed in men than in women, they contribute to a higher risk of developing COPD and heart disease. COPD is also an independent risk factor for pneumonia in patients with DM [23]. In addition, men are more likely to experience occupational exposure to pathogens such as *Klebsiella*, *Streptococcus pneumoniae*, and *Chlamydia pneumophila* [24]. Furthermore, during COVID-19, when there was a significant increase in mortality rates among both sexes, this disparity persisted, with men accounting for a higher percentage of overall mortality. This may be because, compared with women, men are more likely to dismiss the risk of COVID-19 and, as a result, adopt fewer behavioral modifications, such as wearing masks or avoiding contact with people outside the home. It has also been reported that wearing masks is perceived by some men as a sign of “frailty” or “weakness” [25,26]. Notably, essential services, including transportation, law enforcement, emergency response, and industry, have a predominantly male workforce, resulting in a higher risk of exposure to the COVID-19 virus [27]. Furthermore, during hospitalization for COVID-19, poor diabetic control and compliance have been linked to severe pneumonia and poorer outcomes in patients with DM. Specifically, elevated plasma glucose levels are a predictor of fatal COVID-19 in men, but not in women [28]. Men also exhibit a greater inflammatory response in airways, due to increased accumulation of neutrophils and cytokines such as interleukin-8, interleukin-1β, and tumor necrosis factor-α, which likely contributes to the higher incidence and worse outcomes observed in men [29].

On the other hand, females have stronger immunity against viral infections, and this enhanced response can be attributed to increased antibody production driven by hormonal and genetic factors [30-32]. However, there is a more sincere counterpoint to these seemingly rosy figures for women. Compared with men, women with DM type 2 have a higher relative risk of CVD and mortality. Furthermore, young women with type 2 DM are less likely than men to receive the treatment and CVD risk reduction strategies recommended by current guidelines. These guidelines also lack information regarding gender-sensitive prevention and management approaches. Further research into the underlying mechanisms is essential to strengthen the evidence base and improve outcomes moving forward [33].

### Age Differences

The mortality trends by age stratification shown in our study differed significantly among older adults, middle-aged, and younger individuals. One possible explanation for this finding is that older adults with DM are at increased risk for CKD and CVD [34], both of which have been associated with higher mortality in patients with diabetes with pneumonia [35].

Evidence also suggests that multisystem geriatric syndromes are increasingly linked to DM [36]. These syndromes, including falls, functional decline, and delirium, are more common among older adults. They often contribute to disability and lower health-related quality of life, acting as barriers to regular check-ups and follow-ups, and potentially leading to poorer overall health care outcomes [37]. Furthermore, aging, increased levels of HbA_1c_, and the accumulation of glycation end products may heighten susceptibility to infections in older individuals, as these factors are associated with diminished immune function and can contribute to increased morbidity and mortality [38].

DM is associated with a reduced health-related quality of life [38]. Contributing factors include the effects of polypharmacy, which can increase the risk of drug interactions, exacerbate geriatric syndromes, and even lead to death, as well as a greater risk of other chronic conditions and diabetes-specific complications. Additionally, the higher likelihood of older Americans having an annual income below the federal poverty level suggests that many seniors may have a limited capacity to manage DM and its consequences, due to socioeconomic constraints [39,40].

### Racial/Ethnic Disparities

Racial trends in mortality due to DM and pneumonia show the highest mortality rates among NH AI/AN individuals, with Hispanics and NH African Americans occupying the second position at different points in time. A significant factor underlying this trend in NH AI/AN populations is the consistently higher prevalence of DM across all ages and both sexes [39]. This prevalence is nearly 3 times higher than that of US NH White individuals [41,42]. Additionally, AI/AN populations with DM are significantly more prone to CVD, approximately 3-4 times more than those without DM [43]. These populations also tend to have a higher prevalence of multiple risk factors, including tobacco use, DM, hypertension, and dyslipidemia [44]. According to a previous study, 26.7% of AI/AN adults identified as smokers, making tobacco use a major public health concern in this group. Similarly, approximately 10% of individuals aged 12 years and older reported alcohol abuse, the highest rate among all the ethnic groups in the United States [45]. Public health care initiatives targeting these communities, especially those focused on smoking and alcohol use, could help raise awareness. Support groups and addiction clinics may also provide considerable benefit in addressing these issues.

Moreover, socioeconomic factors also contribute to this disparity. In 2010, 23% of AI/AN families had incomes below the poverty line, compared with 16% of the general population [46]. In addition, the environment faced by these individuals is often marked by lower educational attainment and higher stress levels. These factors make it more difficult for certain populations to access and afford health care services necessary to manage complex conditions involving the coexistence of DM and pneumonia [47,48]. Furthermore, a significant increase in mortality was observed among all racial groups during the COVID-19 pandemic, with the effect being particularly pronounced in the AI/AN population. This may be attributed to the greater social vulnerability and exposure to structural racism in these communities, which make them more susceptible to adverse health outcomes and mortality during public health emergencies, such as COVID-19 infections in patients with DM [49]. This situation is further compounded by widespread mistrust of medical institutions, which is more common among Black and AI/AN communities. During the COVID-19 era, this mistrust contributed to vaccine hesitancy, thereby increasing the burden of respiratory infections in these populations [50].

### Urban-Rural Differences

Our study revealed a greater increase in mortality rates in rural areas than in urban areas. This disparity can be attributed to the limited access rural residents have to the specialized health care required to manage DM and its complications [51]. The rural population in the United States also has a relatively high percentage of uninsured individuals [52]. Notably, only 11% of all physicians choose to practice in rural settings [53], leading to a shortage of emergency services, critical care resources, and subspecialty care [54]. To worsen matters, limited transportation options and longer travel times to health care facilities further contribute to the overall problem [55]. Telehealth and mobile health care services have the potential to address some of these deficiencies; we discuss these approaches in greater detail below. Furthermore, rural populations experience a higher prevalence of chronic conditions such as DM, largely due to increased rates of obesity and metabolic syndrome in these areas [56]. In addition, the concentration of unhealthy older individuals in rural communities further increases vulnerability to both DM and pneumonia [57].

Additionally, rural areas have a disproportionately large older adult population, comprising 22% of all older adults in the United States, despite only 15% of the overall US population residing in these regions [58]. As older individuals are at increased risk of developing DM and pneumonia, their growing presence in rural areas may help explain the observed mortality trends. Moreover, a high risk of pneumonia in rural areas has been linked to increased indoor and outdoor air pollution, as well as agricultural waste, which releases reactive nitrogen species and fine particulate matter, both of which adversely affect respiratory function [59]. Proximity to poultry farms and other agricultural settings has also been associated with an imbalance in the microbial composition of the respiratory tract, increasing susceptibility to pneumonia [60]. Finally, rural health systems were comparatively slower in responding to the COVID-19 pandemic. Public health measures such as mask wearing and lockdowns had a limited impact on behavior in these communities [61].

### Geographical Trends

In terms of regions, the West and South recorded the highest mortality rates. According to census reports, Southern states such as Virginia and Florida have median ages at or above 60 [62]. As previously established, older individuals are more predisposed to DM and its complications, which may help explain the elevated mortality in this region. Similarly, African Americans are more heavily concentrated in the Southern states [63], and we have shown that this population experiences higher mortality rates due to DM and pneumonia. The increased mortality rates observed in the West may be attributed to the rising populations of AI/AN individuals in states such as California, Alaska, and Arizona [64]. Given that AIs/ANs exhibited the highest mortality rates in our study, this demographic shift could be a contributing factor to the trend. However, literature on regional mortality trends due to DM and pneumonia remains limited, and further research is needed to better understand this variable.

### Mortality Stratified by Type of Diabetes Mellitus

The significant decline in AAMRs among patients with type 1 DM from 1999 (9.2) to 2015 (1.4) strongly suggests improved use of insulin pumps, continuous glucose monitors, mobile apps, and advanced insulin therapies, all of which have contributed to better glycemic control [65]. Moreover, improved influenza and pneumococcal vaccination coverage, along with early management of respiratory infections in younger populations with diabetes, has further contributed to reduced mortality over time [5]. However, the reversal of this trend after 2015, with AAMR peaking at 3.2 in 2021 before a slight decline in 2022, warrants attention. The rise in pneumonia-related mortality among patients with type 1 DM between 2015 and 2021 may be attributed to an increase in adult-onset type 1 DM, inadequate adult vaccination coverage, and poor glycemic control during transitional phases in adolescents and young adults. The COVID-19 pandemic further exacerbated these risks through immune dysregulation, altered angiotensin-converting enzyme 2 expression, and health care disruptions, which may have delayed appropriate management [66]. The decline observed after 2022 is likely attributable to the effectiveness of COVID-19 vaccines.

Contrastingly, in type 2 DM, AAMRs remained high but relatively stable from 1999 (18.8) to 2017 (19.4), suggesting a plateau in the effectiveness of interventions—a balance between improved care and the rising prevalence of diabetes. However, mortality increased significantly after 2017, reaching 62.2 by 2020. After 2017, the disproportionate impact of the COVID-19 pandemic—characterized by immune dysfunction, cytokine storms, hospital overload, and disruptions in diabetes care—combined with rising obesity, advanced age, and comorbidities to drive a dramatic increase in pneumonia-related deaths among patients with type 2 DM [67]. Polypharmacy, frailty, reduced physical activity, and mental health issues further compromised glycemic control. The subsequent decline after 2020 likely reflects the impact of targeted public health initiatives, improved treatment guidelines, the expansion of telemedicine, and prioritized COVID-19 vaccination [68].

### COVID-19 Pandemic–Driven Reversal of Mortality Gains

The pandemic reversed 2 decades of progress, erasing 17 years of declining mortality gains as overall rates rebounded to near-1999 levels by 2022. Adults aged 65 and older were most severely affected, with AAMRs rising sharply from 206.9 in 2017 to 371.3 in 2022, reversing prior improvements. Although men consistently had higher death rates, women experienced a steeper increase during the pandemic (APC 24.88 in males vs 27.60 in females). Rural communities—already at a disadvantage—experienced catastrophic spikes (APC 64.42 after 2018), while AI/AN populations faced the most severe inequities. The West and South bore the highest mortality burdens, likely due to their higher proportions of aging populations and AI/AN residents. Crucially, mortality related to type 2 diabetes and pneumonia—previously stable—surged during the pandemic, with AAMRs peaking at 62.2 in 2020. This spike reflects the lethal synergy between COVID-19 and metabolic dysfunction, exacerbated by disrupted care and widespread comorbidities.

### Future Interventions

A number of interventions can be implemented in the future to improve control and prevention. The first and foremost is greater vaccination coverage. Influenza vaccines, such as the 23-valent pneumococcal polysaccharide vaccine (PPV23) and the 13-valent pneumococcal conjugate vaccine (PCV13), have significantly improved the prognosis of patients with pneumonia with increased risk factors such as DM [69,70]. Second, controlling plasma glucose levels is essential for reducing the risk of pneumonia in patients with DM. Therefore, the next key intervention is improving glycemic levels and their control through the use of noninvasive glucose monitoring systems [71]. Telehealth opportunities and improving health care equity can help reduce rural-urban disparities in trends [72]. To enhance health literacy among the AI/AN population, the National Heart, Lung, and Blood Institute has developed the Honoring the Gift of Heart Health curriculum [73]. Health literacy plays an essential role in addressing medical mistrust within ethnic communities. The development of specific and targeted programs, such as the National Diabetes Education Program (to provide diabetes education for various audiences), the Chronic Kidney Disease Initiative, and the Diabetes Prevention Program (to improve lifestyle factors among patients with DM), has the potential to significantly reduce mortality trends across all age and racial groups.

### Limitations

There are several limitations to this study. First, reliance on death certificates and ICD codes may have resulted in both unintentional misreporting and underreporting of DM and pneumonia as causes of death. Cause-of-death reporting can be inconsistent; in particular, diabetes is frequently underreported on death certificates. A CDC analysis found that only 41% of decedents known to have diabetes had it recorded on their death certificate [74]. Therefore, the use of death certificates likely underestimates the true incidence of pneumonia-related deaths in individuals with diabetes and may bias results toward cases where physicians explicitly linked the 2 conditions. Furthermore, the potential impact of changes in coding practices (especially the shift in coding pneumonia vs COVID-19 in 2020 and beyond) cannot be ruled out. Second, variables such as vital signs, laboratory findings, and genetic data were not available, even though they are known to be important in understanding the extent of the disease. These omissions mean that potential confounders (eg, poverty, which correlates with both diabetes prevalence and pneumonia outcomes) could not be adjusted for, and causal inferences remain speculative. The study design is purely descriptive and therefore cannot determine why certain groups experience higher mortality—for instance, whether this is due to higher prevalence of diabetes, differences in health care, or other risk factors. Third, AAMRs reflect population-level burden but conflate DM prevalence with case-fatality risk. Future research would benefit from examining mortality rates specifically among individuals with diabetes, or determining the proportion of pneumonia deaths that occur in those with diabetes, to better assess the risk to patients with diabetes versus the broader population-level burden. Fourth, joinpoint analyses were conducted across multiple subgroups without formal correction for multiple comparisons. Although the major trend inflection observed around 2016‐2018 was consistent across most subgroups, smaller or borderline changes in individual subgroups should be interpreted with caution. Identical settings were applied across subgroups, with weighted BIC selecting 2 joinpoints in each case to enable comparability; however, this uniform approach may underfit or overfit trends specific to individual subgroups. Formal sensitivity analyses to explore alternative parameter choices (eg, minimum observations between joinpoints, maximum number of joinpoints) were not conducted due to resource constraints; this may have affected the detection of shorter or additional trend segments. Residual diagnostics for the overall model indicated a Durbin-Watson statistic of 1.62, suggesting mild positive autocorrelation. While basic visual checks did not reveal extreme systematic departures, residual correlation cannot be entirely ruled out and represents a potential limitation. Fifth, data on the efficacy of interventions are limited, as information on the impact of medical therapies and treatments for DM and pneumonia is not available. Additionally, baseline characteristics such as atherosclerosis, atrial fibrillation, ischemic conditions, or other coexisting infections, which may significantly contribute to mortality in patients with DM or pneumonia, are also missing. Finally, datasets concerning socioeconomic factors and educational attainment are unavailable; these variables may influence access to care and health outcomes, further limiting the study’s scope.

### Conclusion

Mortality trends among patients with DM and pneumonia varied across all demographic factors. Although an overall decline in mortality rates was observed across most groups, a significant increase occurred during the COVID-19 period. The highest mortality rates were reported among older individuals (65-86+ years), males, and NH AIs. Additionally, nonmetropolitan areas, especially in the West region, experienced the highest mortality. Therefore, glycemic control in all patients with DM is essential, whether through monitoring systems or preventive programs. There is a pressing need to allocate sufficient resources to underserved regions and overlooked populations to improve their mortality outcomes. Longitudinal surveillance and tailored interventions (eg, telehealth, mobile clinics) targeting high-risk populations are urgently needed to mitigate disparities.

## Abbreviations

AAMR: age-adjusted mortality rate
AI: American Indian
AN: Alaska Native
APC: annual percentage change
BIC: Bayesian information criterion
CDC WONDER: Centers for Disease Control and Prevention Wide-ranging Online Data for Epidemiologic Research
CKD: chronic kidney disease
COPD: chronic obstructive pulmonary disease
CVD: cardiovascular disease
DM: diabetes mellitus
ICD-10: International Statistical Classification of Diseases and Related Health Problems, 10th Revision
NH: non-Hispanic
STROBE: Strengthening the Reporting of Observational Studies in Epidemiology
